# Historical development of mesenteric anatomy provides a universally applicable anatomic paradigm for complete/total mesocolic excision

**DOI:** 10.1093/gastro/gou046

**Published:** 2014-07-17

**Authors:** Rishabh Sehgal, J. Calvin Coffey

**Affiliations:** ^1^Centre for Interventions in Infection, Inflammation and Immunity (4i), Graduate Entry Medical School, University of Limerick, Ireland and ^2^Department of Surgery, University Hospitals Group Limerick, Limerick, Ireland

**Keywords:** mesocolon, mesenteric excision, complete mesocolic excision, Toldt’s fascia

## Abstract

Although total mesorectal excision has now become the ‘gold standard' for the surgical management of rectal cancer, this is not so for colon cancer. Recent data, provided by Hohenberger and West *et al.* and others, have demonstrated excellent oncological outcomes when mesenterectomy is extensive (as is implicit in the concept of a ‘high tie') and the mesenteric package not violated. Such studies highlight the importance of understanding the basics of the mesenteric organ (including the small intestinal mesentery, mesocolon, mesosigmoid and mesorectum) and of abiding to principles of planar surgery. In this review, we first offer classic descriptions of the mesocolon and then detail contemporary thinking. In so doing, we provide an anatomical basis for safe and effective complete mesocolic excision (CME) in the management of colon cancer. Finally we list opportunities associated with the new anatomical paradigm, demonstrating benefits across multiple disciplines. Perhaps most importantly, we feel that a crystallized view of mesenteric anatomy will overcome factors that have hindered the general uptake of CME.

## INTRODUCTION

In the recently published article entitled *Re-examination of the standardization of colon cancer surgery* [1], the authors comprehensively reviewed the evidence base for complete mesocolic excision (CME) (also known as total mesenteric excision) [2, 3]. They should be commended for critically appraising the Standard for the Diagnosis and Treatment of Colorectal Cancer (2010), issued by the Chinese Ministry of Health [4], and for re-examining the core principles of General Rules for Clinical and Pathological Studies on Cancer of the Colon, Rectum and Anus (seventh edition) [5]. Importantly, they have highlighted several issues that continue to hinder the overall acceptance of complete/total mesenteric excision.

Although total mesorectal excision (TME) has become the ‘gold standard’ for the surgical management of rectal cancer, this has not occurred for colon cancer [6, 9]. In describing TME, Heald *et al.* provided a cogent anatomical basis reflected in rich terminology, such as the ‘holy plane’ [10]. Unfortunately, complete/total mesenteric excision was not preceded by a similar anatomical description, and confusion has persisted over the gastrointestinal planes exploited. Recent data provided by Hohenberger and West *et al.* have demonstrated excellent oncological outcomes when mesenterectomy is extensive (as is implicit in the concept of a ‘high tie’) and the mesenteric package not violated [11, 12]. These and more recent studies highlight the importance of understanding the basics of the mesenteric organ (including the small intestinal mesentery, mesocolon, mesosigmoid and mesorectum) and abiding to principles of planar surgery [9, 13–18].

Unfortunately, the first contemporary anatomical characterization of the mesenteric organ was published after the above manuscripts. Culligan *et al.* [19] conducted the most recent formal appraisal of mesenteric organ anatomy and comprehensively refuted classical thinking as laid down by Sir Frederick Treves in 1885 [20]. The latter is fundamentally based on the perception of the human mesentery as a fragmented structure with complex relationships, and was indoctrinated into mainstream surgical, anatomical, and embryological literature over the past century. In contrast, contemporary appraisals have confirmed that the mesenteric organ is in fact a continuous structure from the duodeno-jejunal flexure to the mesorectum [19, 21–23]. This greatly simplified concept has generated several opportunities for further study across numerous basic and applied sciences.

In this review, we first outline the classic descriptions of the mesocolon and then detail contemporary thinking based on recently performed appraisal of the mesocolic organ. In so doing, we provide an anatomical justification for performing safe and effective CME for the management of colon cancer. Finally we list opportunities associated with the new anatomical paradigm, demonstrating benefits across multiple disciplines.

## ANATOMY OF THE MESOCOLON: HISTORICAL PERSPECTIVE

**Figure 1.**
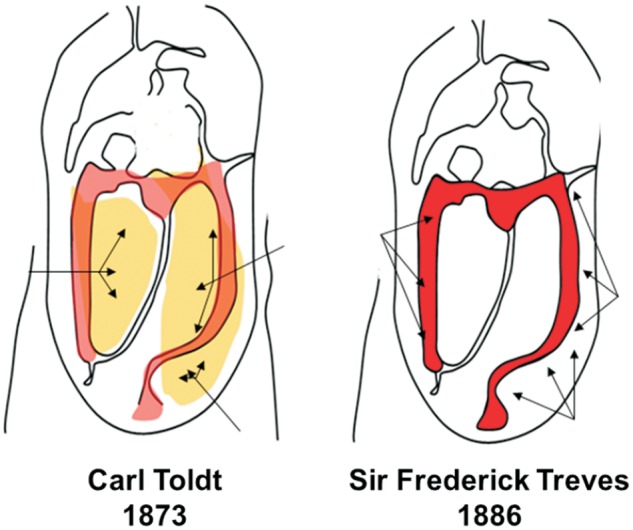
**Left: **the mesocolic attachment to the posterior abdominal wall is demonstrated as depicted by Carl Toldt, with the arrows indicating the extent of the attachment. **Right: **the mesocolic attachment as depicted by Frederick Treves, with the arrows indicating the extent of the attachment.

The classical anatomical description of the mesocolon originated from observations made by Sir Frederick Treves in 1885 [20]. Treves was a British surgeon in the late 19th and early 20th centuries. He performed one of the earliest appendectomies in 1888 and was surgeon to both Queen Victoria and King Edward VII [24]. Treves studied the anatomical arrangement of the human intestinal canal, peritoneum and mesentery. He delivered his case series of 100 cadaveric dissections at the Royal College of Surgeons in England and noted that there was neither an ascending nor a descending mesocolon in approximately 50% of cadavers studied. In 22 cadavers, there was a descending mesocolon, but no trace of a corresponding fold on the contralateral side. In 14 subjects, there was a mesocolon to both the ascending and descending segments of the bowel; in the remaining twelve bodies there was an ascending mesocolon, but no corresponding fold on the left side. Treves concluded that a mesocolon would therefore be present in 36% and 26% of adults on the left and right hand side, respectively (Figure 1) [20].

Treves’ descriptions of the mesocolon laid the foundation for anatomical, embryological and surgical textbooks, and have been widely reproduced in the literature to the present day; for example, the 15th edition of Gray’s Anatomy (1901) quotes Treves’ findings verbatim [15]. More recently the 40th edition of Gray’s Anatomy (2008) similarly suggest “The ascending colon possesses a narrow mesocolon for part of its course in up to one-third of subjects” [26]. Further, the authors suggest that the ascending colon is a “… retroperitoneal structure covered anteriorly and on both sides by peritoneum” [26]. Similarly, Last’s Anatomy suggests that for the ascending colon “… the original embryonic mesentery is retained in about 10% of adults”, while the descending colon “… in the whole of its course is plastered to the posterior abdominal wall by peritoneum (like the ascending colon), though a mesentery is present in about 20% of adults” [27].

The identification of a right/left mesocolon in the adult is thus frequently depicted as anomalous, rather than accepted as an anatomical norm. Adams described the persistence of an ascending mesocolon in the adult as being “as abnormal as a cleft palate” [28]. At best, the ascending and descending portions of the mesocolon are described as “secondarily retroperitoneal” following “fusion” of the associated mesentery with the underlying retroperitoneum [29].

It often comes as a surprise to anatomists that the surgical approach to the mesocolon has relied upon the persistence of all portions of the mesocolon into adulthood. In conducting a total or partial right mesocolectomy (in which colectomy is implicit) the entire right mesocolon is firstly mobilized then resected. Similarly, in conducting a left-sided mesocolectomy (partial or total), the entire left mesocolon must first be mobilized and vessels ligated and then resected. In total mesocolectomy, the entire colon and associated mesocolon are removed *en bloc* [23, 30].

Interestingly, in 1879, Carl Toldt made prescient observations prior to those of Treves when he described persistence of the mesocolon into adulthood (Figure 1). Toldt was an Austrian anatomist who was professor of anatomy in Prague and Vienna. He published his account pertaining to the structure and development of the human mesentery in 1879. In his anatomical textbook *An Atlas of Human Anatomy for Students and Physician*, Toldt illustrates persistence of the entire mesocolon into adulthood [31]. Toldt also identified a distinct fascial plane between the mesocolon and the underlying retroperitoneum, formed by the fusion of the visceral peritoneum of the mesocolon with the parietal peritoneum of the retroperitoneum (Toldt’s fascia). It is noteworthy that the original English translation of Toldt’s findings refers to a disparity between German and English terminology related to the mesocolon. In England, the term *mesentery* came to denote the peritoneal folds suspending freely mobile portions of the alimentary canal, and thus could not be reconciled with Toldt’s description of the mesocolon. The existence of a distinct descending mesocolon (in the English sense of the term) was rare. It is likely therefore that this semantic restriction contributed to the misconceptions regarding the non-mobile portions of the ascending and descending mesocolon that were not retained into adulthood [32].

Toldt’s findings were repeated in 1942 by Edward Congdon, who demonstrated that the right and left mesocolon persist into adulthood, universally remaining separate from the retroperitoneum [33]. There was little uptake of this concept until 1986, when Dodd, a radiologist, indicated that, unless the mesocolon remained an extra-retroperitoneal structure (i.e. separate from the retroperitoneum), the radiological appearance of the mesentery and peritoneal folds could not be reconciled with actual anatomy [34]. Again, there has been limited acceptance of this concept to the present day, since radiologists continue to describe the mesentery and peritoneal folds as a difficult field [35]. Interestingly, the emergence of high-magnification and -resolution laparoscopic surgery saw the re-introduction of terminology such as ‘mesocolon’ and ‘Toldt’s fascia’ and led to a resurgence in interest in surgical anatomy in general. Whilst this commenced with a clarification of mesenteric organ anatomy (see below), it subsequently led to a clarification of peritoneal folds, *appendices epiploicae*, the mesoappendix, congenital adhesions and attachments of the greater omentum [23, 36].

## THE MESOCOLON: CONTEMPORARY WORK

In 2012 our group prospectively characterized mesocolonic anatomy in patients who underwent a total mesocolic excision (i.e. the entire colon and associated mesocolon are resected *en bloc*) [19]. In so doing, several anatomical findings emerged that had not previously been documented. In their cohort of 109 patients, the mesocolon was continuous from ileocaecal to rectosigmoid level. A mesenteric confluence occurred at the ileocaecal and rectosigmoid junction, as well as at the hepatic and splenic flexures. Each flexure (and ileocaecal junction) was a complex of peritoneal and omental attachments to the colon and centred on a mesenteric confluence. Moreover, the proximal rectum originated at the confluence of the mesorectum and mesosigmoid. A plane occupied by Toldt’s fascia separated the entire adherent mesocolon from the retroperitoneum [19].

These findings therefore provided a rationalization of the surgical, embryological and anatomical approaches to the mesocolon. Shortly after, we determined the histological and electron-microscopic appearance of the mesocolon, fascia, and retroperitoneum, prior to and after colonic mobilization [21]. In 24 cadavers, tissue samples were taken from all aspects of the large bowel (ascending, transverse, descending and sigmoid mesocolon) and stained with hematoxylin and eosin, Masson trichrome and podoplanin. The microscopic structures of the mesocolon and associated fascia were consistent from ileocecal to mesorectal level. A surface mesothelium and underlying connective tissue were evident throughout. Fibrous *septae* separated adipocyte lobules within the body of the mesocolon. Where apposed to the retroperitoneum, two mesothelial layers separated mesocolon and underlying retroperitoneum. A connective tissue layer occurred between these (i.e. Toldt’s fascia). Lymphatic channels were evident both in mesenteric connective tissue and Toldt’s fascia. We demonstrated that, during complete/total mesocolic excision, the mesocolon and underlying fascia (Toldt’s fascia) remained intact and contiguous. In other words, the interface or plane (i.e. meso- and retrofascial planes) utilized in this surgery is formed by these contiguous structures [21].

## COMPLETE/TOTAL MESOCOLIC EXCISION

The standardization and clear anatomical conceptualization of TME have greatly aided in the standardization of rectal cancer. Unfortunately, survival rates for colon cancer have remained static, unlike that for rectal cancer. To date, rates of local and distal recurrence vary considerably between treatment centres. A recent systematic review, that incorporated 21 predominately retrospective studies and evaluated the evidence regarding oncological outcomes, morbidity and mortality after CME or extended lymphadenectomy for colon cancer, reported inconsistent data amongst the studies. Only five of these reported 5-year local recurrence data, which revealed weighted mean local recurrence-, 5-year overall- and disease-free survival rates of 4.5% (range 2–7.8%), 58.1% and 77.4%, respectively [37].

Planar dissection, combined with a high vascular tie, aims to produce a specimen with intact fascial layers whilst simultaneously maximizing lymph node yield. Hohenberger reported an R0 resection in 97% of patients who underwent surgery with curative intent. Application of this technique reduced local 5-year recurrence rates from 6.5% to 3.6%, whilst, in patients resected for cure, cancer-related 5-year survival rates increased from 82.1% to 89.1% [11]. Unfortunately, in original and contemporary descriptions of CME, current appraisals of mesenteric organ anatomy are largely ignored. As a result, inaccurate terminology, such as ‘visceral and parietal fascia’, continues to be utilized. Moreover, the concept of mesenteric contiguity, as well as the associated surgical planes and peritoneal folds, remain entirely ignored.

West *et al.* adopted the CME principles of avoidance of mesocolic disruption, further examining the relationship between the plane of surgical resection and survival in colon cancer [12]. In a cohort of 399 colon cancer cases, the plane of surgery was associated with the *muscularis propria* in 95 (24%) of specimens, intra-mesocolic in 177 (44%), and mesocolic (i.e. not disrupting the mesocolon) in only 127 (32%). On univariate analysis, they demonstrated a 15% overall survival advantage at 5 years when surgery was performed with mesocolic *vs*. intramesocolic or *muscularis propria* plane approaches [12]. Unfortunately, the implications of mesocolic contiguity were not discussed; for example, as the mesenteric organ spans the gastrointestinal tract from duodenojejunal to mesorectal level, then intramesocolic surgery is unavoidable. In dividing vessels contained within the mesocolon, and in dividing the mesocolon up to the gastrointestinal level, it is not possible to remain entirely mesocolic.

Several authors have recently demonstrated improved oncological outcome with complete/total mesenteric excision, but none has detailed the precise anatomical basis of the techniques involved. Bokey *et al.* compared historical data (from 1971-1979) following the establishment of a dedicated colorectal unit (1980-1995) and introduction of a standardized technique for colectomy. Their approach was based on “… precise dissection along anatomical planes facilitating an operation that will not compromise or breach the facial envelope of the colon and its mesentery”. In analysis of this series of 867 patients, the authors demonstrated that the overall 5-year survival rose from 48.1% to 63.7% [9]. Despite their emphasizing the importance of anatomical dissection, at no point is there a description of mesocolic contiguity and its technical implications, nor is there comment on the complex of peritoneal folds that enwrap the gastrointestine itself and associated mesentery.

Similar studies by Storli *et al.* and Galizia *et al.* demonstrated improvements in oncological outcomes, such as local recurrence and overall survival rates [17, 18]. In these studies, the importance of anatomical dissection is repeatedly emphasized but at no point is there a detailed appraisal of mesenteric organ or peritoneal fold anatomy.

Furthermore, the emergence of laparoscopic high-magnification and resolution-based techniques has highlighted the importance of adhering to fundamental surgical tissue planes in order to perform a safe and effective CME. The latest systematic review focusing on complete mesocolic resection and extended lymphadenectomy for colon cancer reported a 15.5% (*n* = 408) frequency of patients who underwent laparoscopic CME as outlined in nine studies [37–48]. Although overall outcome data in the laparoscopic group were comparable with those in the ‘open’ cohort, all included studies were case studies of prospective or retrospective nature, and there was no Level 1 evidence from a randomized, controlled trial, thereby making it difficult to draw any firm conclusion pertaining to laparoscopic CME [37].

## SUMMARY

To date all manuscripts describing complete/total mesocolic excision have emphasized the importance of anatomical dissection. All emphasize the importance of achieving a high tie, as well as extensive and intact mesenterectomy. However, in all, readers are required to interpolate progression from entering an undisturbed abdomen, to getting into the mesofascial plane, mobilizing along this plane, dividing vessels within the mesocolon and mobilizing each flexural complex (comprised of mesocolic confluence, gastrointestinal tract and peritoneal fold). Given the importance of anatomical rationale in the standardization of rectal cancer excision, it behoves the surgical community to appraise colonic and small-intestinal mesenteric anatomy, with the same vigour.

## CONCLUSION

For over a century, mesenteric anatomy has been universally depicted in an inaccurate manner. Recent observations confirm a simpler and continuous structure from duodenojejunal flexure to mesorectum. This appraisal aids in understanding the peritoneal folds, adhesion anatomy, the origin of the meso-appendix and the attachment of the greater omentum. From a technical perspective, the new paradigm permits safer surgery that achieves an extensive and intact mesenterectomy. The resultant surgery is nearly bloodless, with minimal rates of injury to adjacent organs, and is readily transmissible to the trainee gastroenteric surgeon. From an oncological perspective, the intact and extensive mesenterectomy achieves optimal patient-related outcomes.

**Conflict of interest:** none declared.
